# App-Supported Promotion of Child Growth and Development by Community Health Workers in Kenya: Feasibility and Acceptability Study

**DOI:** 10.2196/mhealth.6911

**Published:** 2017-12-05

**Authors:** Alastair van Heerden, Debjeet Sen, Chris Desmond, Julia Louw, Linda Richter

**Affiliations:** ^1^ Human Sciences Research Council Pietermaritzburg South Africa; ^2^ Medical Research Council / University of the Witwatersrand Developmental Pathways for Health Research Unit Department of Paediatrics, Faculty of Health Science University of the Witwatersrand Johannesburg South Africa; ^3^ PATH Johannesburg South Africa; ^4^ University of the Witwatersrand Johannesburg South Africa; ^5^ Assistive Technologies in Autism and Intellectual Disability National University of Ireland Galway Ireland; ^6^ Department of Science and Technology–National Research Foundation Centre of Excellence in Human Development University of the Witwatersrand Johannesburg South Africa

**Keywords:** child health, child development, monitoring and evaluation, parent support, mHealth

## Abstract

**Background:**

Early childhood is a critical phase of development. In low resource settings, monitoring this stage of development and providing appropriate and timely feedback is a challenge. Community-based service providers play a key role in promoting early childhood development in areas where government services are weak. These community-based service providers are also tasked with the collection of monitoring and evaluation data for donors and local government. Usually, collection of these data aims to provide accountability, learning, and correction leading to improvement. However, such data is rarely used beyond the accountability stage.

**Objective:**

The purpose of this study was to test the feasibility and acceptability of the Information for Action (IFA) mobile phone app. The IFA app was designed for use by community health volunteers (CHVs), and repackages routinely collected data about children into useful, offline decision support for caregivers and program managers.

**Methods:**

The IFA app was tested with a convenience sample of 10 CHVs in West Katweng’a, a sublocation of Rarieda subcounty in western Kenya. CHVs used the IFA app for 5 months as part of their regular home visits to households containing children aged 0 to 5 years, after which a qualitative assessment of the app was conducted. A total of 16 caregivers who received services from the CHVs were randomly selected to participate in 1 of 2 focus group discussions about their experience.

**Results:**

The app was reported to help facilitate interactive dialog between CHVs and caregivers, leading to improved quality of home visits. Caregivers described the app as shifting the relationship from feeling harassed by CHVs to experiencing genuine interest from CHVs. CHVs reported feasibility challenges primarily related to infrastructure. The limited battery life of mobile phones combined with the lack of readily available electricity made it difficult to keep the phones charged. CHVs reported initial anxiety as first-time mobile phones users, including concerns about using the IFA app. With time, increased levels of confidence were seen.

**Conclusions:**

Acceptability was high with both CHVs and caregivers, who reported an improvement in their client-provider relationship. A number of feasibility challenges were experienced.

## Introduction

### Background

Early childhood is a critical period in human development [1]. Strong causal evidence suggests that delayed or disrupted early development has long-term negative consequences for children. Impoverished nutritional, emotional, and cognitive circumstances result in risks for ill health, as well as social and psychological difficulties in adulthood [2]. In response, increased efforts are being carried out globally to protect and promote children’s early experiences. These initiatives, aimed at supporting early development, typically take place at health facilities, early child development centers, or through community health volunteers (CHVs) [3,4]. The role of CHVs varies, but typically they are responsible for strengthening the links between the community and health services. Primary responsibilities of CHVs in Kenya include the identification and registration of pregnant women and their young children, assisting in the registration of births, monitoring child growth and vaccination schedules, and reporting notifiable diseases and deaths [5].

Community and home visitors can play a critical role in addressing gaps in the coverage of health services by relaying health education messages, conducting basic health assessments, and providing referrals. However, in reality, they seldom have the tools, support, and feedback required to carry out these functions. Even when data are immediately available, for example, when measuring a child’s height or weight, CHVs do not traditionally have the knowledge or skill to interpret the measurements and data in relation to the child’s age or gender, nor do they necessarily have training in what feeding, nutrition, and health care recommendations to make on the basis of the measurement [6]. In addition, their core function ends up shifting away from the above, primarily toward data collection for donors and government, with no clear benefit perceived by CHVs or families. Under these conditions data quality suffers, further reducing the likelihood that data will be able to be used meaningfully or for quality improvements [7]. A key challenge identified in many programs (both governmental and nongovernmental) is the almost exclusive focus on collecting data on field activities and outputs and sending this information upward to governmental and nongovernmental stakeholders—often located at a distance from the data collection sites. The underutilization of monitoring and evaluation data is generally attributed to top-down designs for routine reporting rather than a focus on learning and improvement of existing systems [5]. There are usually few systems in place which would enable front-line workers and their supervisors to use the data they collect to improve their performance.

In light of the above, this paper reports on a field test of the Information for Action (IFA) mobile phone app developed by a team at the Human Sciences Research Council (HSRC) in South Africa. The app was designed to use CHV data collection activities as an opportunity to provide useful information to families and caregivers about their children’s development.

### Information for Action App

With a view to transforming some of the challenges outlined above, we prototyped and developed an easy-to-use mobile phone IFA app that can simultaneously collect data on growth and child development and provide tailored information and targeted messages to parents and other caregivers, health and social community-based service providers, workers and volunteers, case managers, program managers, and funders.

Harnessing mHealth solutions in the support of maternal and child health is not a new idea. Launched in February 2010, Text4baby *,* for example, has gained wide media attention, winning awards from the United States Office of Science and Technology in 2010 and the Public Relations Society of America in 2011. The Text4baby program uses the health belief model to support mothers through prenatal care text messages [8]. More recently, a consortium of partners including Johnson & Johnson, United States Agency for International Development, United Nations Foundation, and the mHealth Alliance created an alliance to improve maternal health by leveraging mobile technology to deliver health information to mothers in India, South Africa, and Bangladesh [9]. A number of mobile phone–based data collection apps are also available that facilitate the collection of data in low-resource settings [10-13]. A multitude of studies review and test mHealth apps designed to collect health data and provide feedback for the self-management of chronic disease [14-17].

A review of mHealth apps in low- and middle-income countries (LMICs) found that the majority of apps still focus on only one of the following areas: data collection, education, communication, or information sharing [18]. Apps targeting LMICs that integrate multiple functionalities do exist, but resource-limited environments present an additional challenge with the low availability of high-speed, stable, mobile Internet [19]. The implication of this is that all data processing and decision support needs to reside on the device rather than on a central server. This study adds to this growing body of literature by examining the acceptability and feasibility of the maternal and child health IFA app, which was designed specifically for women and young children living in LMICs.

### Participants

The IFA app was piloted in the West Katweng’a sublocation of Rarieda Subcounty in western Kenya. The community-based organizations (CBOs) in this sublocation had 10 CHVs under their supervision. All 10 were invited to participate in the study. No new CHVs were hired or trained for the purposes of the field test. All CHVs were already working in the area and were familiar with the terrain and households in their catchment area. No direct financial incentives were offered for participation. However, the CHVs participating in the field test were allowed to purchase the phones used in the study by paying a price that was well below market value (4500 Kenyan Shillings or US $50 at the time of the study). In the local context, this was still a high price, as CHVs are currently (2017) paid 2000 Kenyan Shillings a month for their work in the same sublocation. A total of 16 caregivers, selected at random from among the households visited by the CHVs, were also contacted after the study period and invited to participate in 1 of 2 caregiver focus group discussions.

## Methods

### Information for Action App

The IFA app includes a child growth and development assessment function, which produces useful information that is provided to parents, caregivers, and front-line home visitors providing community-based services. At the same time as pushing data for action down to where it matters most, the information is also aggregated and stored on a central server where it can be used for timely training and supervision of community-based service providers and adjustments to program implementation.

**Figure 1:**
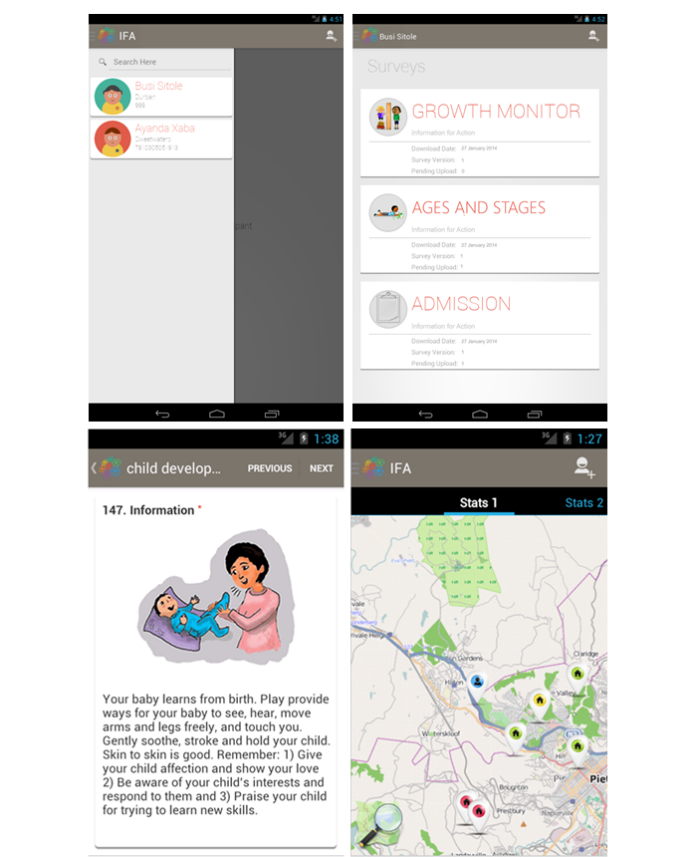
From top left clockwise, the screen shots illustrate (A) selecting a participant, (B) selecting an assessment domain, (C) community-based mapping, and (D) caregiver feedback.

For illustrative purposes, we selected 2 critical domains for monitoring young children’s development—growth (height and weight), a proven measure of both child well-being and development [14], and a measure of psychosocial development (Figure 1). The World Health Organization (WHO), which sets guidelines on child development milestones, provides standardized growth charts by age for gender [15]. These standards were embedded in the IFA app for reference. Although specialized equipment is needed for gold-standard growth monitoring, an inexpensive bathroom scale that can be calibrated and a stadiometer (or measuring tape) is used in this context of home- and community-based service delivery.

Psychosocial development is assessed with the Ages and Stages Questionnaire, Third Edition (ASQ-3), a widely used early childhood development tool that was tested for applicability in the context [16]. The ASQ-3 is an easy to use, reliable, and valid screening instrument to identify potential developmental delays among children aged 2 months to 5 years. It taps into 5 domains of children’s development: communication, gross motor, fine motor, problem solving, and personal-social, with each domain consisting of 6 developmentally appropriate items at 21 time points. Caregivers respond to each item by selecting yes, sometimes, or not yet (see Multimedia Appendix 1).

Using the IFA app, a community-service provider inputs identifying information about a child which can be anonymized at any level of data output. For example, name, photograph, date of birth, gender, location, and other required social and demographic information would be added. Age-appropriate items and reference standards are retrieved from the IFA app database, and the items are displayed for completion by the CHV and caregiver. Collected data are compared to norms by the app, and immediate feedback is provided to caregivers and parents about their child’s growth and development using simple messages and diagrams (for example, a range of smiley faces that indicate good vs potentially problematic development). Feedback is then generated in the form of simple, targeted counseling messages relevant to the specific child. The messages were extracted from international feeding guidelines based on recommendations by the Indian Academy of Pediatrics [17] and the WHO–United Nations Children’s Fund Care for Child Development Package [18] (see Figure 1 D and Multimedia Appendix 1). Historical information for the child is stored, and longitudinal assessments are available to CHVs to track and demonstrate progress through time. Additional information such as a map of the households under the care of the CHV and an indication of the length of time since their last visit are also available (Figure 1 C).

### Procedures

The field test used 14 mobile phones donated by Google, running on the Android platform. Ten phones were provided to the CHVs for implementation during the field test, and 4 were given to their supervisors. For the purpose of the field test, the app contained 2 child development tools. The first tool was based on a modified version of the ASQ, and the second included 9 questions based on indicators collected by CHVs in Kenya as part of routine household visits (Multimedia Appendix 2).

A training-of-trainers approach was adopted to train the CHVs. First, a team from the HSRC met with and explained the study and IFA app to the Rarieda District Health Management Team (DHMT) to build their capacity as trainers. Thereafter, 3 members of the Rarieda DHMT and the Community Health Extension Workers (CHEWs) of West Katweng’a sublocation trained CHVs over the course of 2 days. The training curriculum included instruction on the use of mobile phones and on the specific use of the app. The curriculum also included a mix of both theory and practice sessions, such as role-plays involving CHVs using the app in mock household visits.

The CHVs were then asked to use the app as part of their regular home visits between May and September 2014. Each home visit took between 45 and 60 minutes depending on the amount of feedback that was required. During this 5-month period, the research team made 3 visits to the site in order to provide feedback and support to the CHVs and their managers. An information and communication specialist from the DHMT also volunteered time to troubleshoot any technical issues that emerged during the study. CHVs were expected to visit each household in their catchment area at least once a month. The growth indicator tool was completed at each visit and the ASQ-3 completed on every second visit to allow the child opportunity to progress to the next ASQ age range.

Institutional review board approval was obtained in Kenya from the Ethics Review Committee of the Jaramogi Oginga Odinga Teaching and Research Hospital (accreditation number 01713) and in South Africa through the HSRC’s Research and Ethics committee (REC 22/19/02/2014). All participants were required to provide their written informed consent. If the participant was illiterate, best practice was followed and a trusted literate family member or friend was asked to participate in the consenting process and ensure all information was provided accurately before the participant signed or made their mark.

### Data Analysis

All conversations were audiotaped, transcribed, and translated for thematic analysis [20]. Informed by the literature and the preliminary analysis of transcripts, a coding framework was developed. Following this step, the codes underwent another phase of thematic analysis [21] using Atlas Ti 7.5 (ATLAS.ti Scientific Software Development GmbH) software. Two researchers manually analyzed conversation transcripts. If codes were inconsistent, consensus was reached after reanalyzing the transcripts.

## Results

### Overview

The 10 CHVs collected data on 313 children living in 140 households in Rarieda. Children ranged in age from 2 to 8 years with a mean age of 5 years and 2 months. Just under half (149/313, 47.6%) of the children and the majority of caregivers (123/140, 88.1%) were female. Focus group participant demographics are provided in Table 1.

### Acceptability

#### Training and Uptake

Overall, CHVs and caregivers reacted positively to the app. In particular, CHVs were enthusiastic and excited about the use of both the mobile phone and the app. In the beginning, learning how to use the functionalities of a mobile phone (eg, using a touch screen and selecting apps) proved challenging for several CHVs. Given their initial discomfort with using a mobile phone, CHVs also felt that using the phone for counseling caregivers was slower compared to using paper-based tools. With time and support, CHVs gradually became more confident in using mobile phones. They felt the number of questions provided by the app was optimal, with some respondents mentioning that they would be comfortable with more questions added to the survey. A common perception was that the use of the app reduced the need to carry around heavy counseling materials. Due to its ability to convert collected data into actionable information, respondents felt that the app helped CHVs to remember key messages.

The phone is the way to go, some CHVs used to forget some information that was important and that was supposed to be passed to the caregivers. The phones nowadays have all the information and it is not possible to forget something.

Caregiver, FGD

#### Increased Level of Confidence

The improved quality of home visits together with the possession of a fairly sophisticated phone and the ability of CHVs to use them for their work raised the profile of CHVs within their communities.

It [the app] gives feedback depending on how you talk to the caregiver, it gives you specific response and this has built a lot of confidence.

CHV, FGD

Some caregivers mentioned a change in their own attitude toward home visits.

My attitude changed because now I get their help more than it used to be. One used to harass me so much but now...they ask me questions from the form. I have now loved their work.

Caregiver, FGD

#### Improved Communication and Behavior Change

A consequence of increased caregiver satisfaction and acceptance toward CHV home visits was an increase in self-reported uptake of desired behaviors and practice.

The advice I received from the CHV helped me a lot. I used not to use nets, I used to buy mosquito coils but upon the advice of the CHV I started using nets.

Caregiver, FGD

The question-and-answer format of the app was found to facilitate interactive dialog during home visits.

What I think is that the CHVs go beyond what they want from the caregivers. They can ask how you are doing, how the children are doing.

Caregiver, FGD

This shift toward an interactive dialog held other benefits for caregivers.

I was so happy because it gave me the knowhow; I was enlightened by the data collection process, which was also a learning process.

Caregiver, FGD 2

### Feasibility

Feasibility challenges were experienced. Most had to do with the infrastructure required to keep phones operational and linked to the Internet.

That is a big challenge to us I think, because like since yesterday, I have not been able to charge my phone and that is very difficult. I may just continue asking for help from others who have electricity. Even one of my colleagues has electricity and I just charge there. Our CHEW [Community Health Extension Worker (supervisor)] told us that there is a solar charger for ksh. 900 that we can purchase and use it during data collection; can we be given any support from you guys so that we can buy this charger that can hold power for a long time.

CHV, FGD

While decision support was available offline, CHVs reported experiencing challenges when it came to trying to upload data.

I have enjoyed using this phone, these days I read news over the phone, anything I need I find it online, the other day I got some information from Wangapala in south Nyanza and that was very good to me. I also take photos using this phone and I think it’s a good phone. The negative side of this phone is that, if you carry it like that and you want to use it, it ‘hangs’ a lot and sometime you want to send data and the phone ‘hangs’ a lot.

CHV, FGD

After these initial challenges, the Rarieda DHMT offered their support by providing technical assistance to the CHVs through their information and communication technology (ICT) specialist. The inclusion of specialized support had a significant beneficial impact on the implementation of the field test. CHVs mentioned seeking the ICT specialist’s assistance from time to time to troubleshoot issues with running the phone and the app.

...it was like starting to learn how to walk, but with continuous mentorship they have absorbed how to use the smart phones and my assessment is that they now like to use them.

CHV, FGD

**Table 1:** Demographic details of focus group discussion participants.

Characteristic		CHV^a^ (n=10	Caregiver (n=16)
Age, years, mean (SD)		33 (7.7)	26 (10.1)
**Gender, n**			
	Female	7	13
	Male	3	3

^a^CHV: community health volunteer.

## Discussion

### Principal Findings

The IFA app was developed and piloted as part of an effort to enable community-based program staff to both access the data they collect as part of routine monitoring and provide on-the-spot counseling messages and guidance. This was a significant advancement on the parallel systems present in many of the mHealth apps designed for LMICs at that time (ie, the collection of data and the delivery of messages through short message service). Currently, the app is able to convert growth measurements into field-usable data, provide child development assessments using the ASQ-3, and deliver care and feeding messages appropriate to the assessment. We have also developed field management tools that facilitate improved planning and supervision of the work responsibilities of community-based service providers.

The pilot indicates good reception and use of the app by community-level workers, their supervisors, and the health management team. In addition, qualitative follow-up with a small group of mothers and other caregivers who received home visits during which the app was used reported that they felt they received more targeted assistance and had more confidence in the CHV. Functions of the app that were particularly appreciated by all stakeholders in this field test (beneficiaries, service providers, and supervisors) were the ability of the app to facilitate dialog during home visits and the ability of the app to convert routinely collected data into actionable information and facilitate use of data for decision making at the point of data collection. The app thus addresses common gaps in community health services in low-resource settings. These two functions need to be emphasized in future projects involving the app. These advantages should be weighed against the potential negative consequences of moving data collection to a mobile app. One clear risk is that these data, if stored online, could be accessed, stolen, and leaked or sold. If sensitive data such as the caregiver name, contact number, home Global Positioning System coordinates, and HIV status were included in the dataset, it could be used to extort money from the caregiver. Some feasibility challenges remain, but they are not insurmountable. For example, purchasing solar-powered chargers as a one-off investment can help mobile phones remain consistently charged without having to incur recurring expenses of charging phones at a commercial charging station. Network coverage is improving at a fast pace as demand for and uptake of mobile phones increases in African countries and elsewhere. Also, in resource-poor environments data is becoming cheaper. Further pilot work will address the challenges identified and explore the use of the app by mothers and other caregivers themselves to monitor their own child’s growth and development and to report on indicators tracked by local health and social services.

### Limitations

This study has 3 main limitations. First, the IFA app was only tested for feasibility and acceptability with a small number of CHVs and caregivers. It is not possible to draw broad conclusions on how other CHVs in Kenya would view the app. Second, the donated devices were a few years old, and the poor battery life experienced may partially be due to this fact. Testing the app using recently purchased mobile phones may improve the user experience. Finally, longitudinal data was not available to support the qualitative findings of improved counseling and subsequent outcomes.

### Conclusion

Data collection instruments are often designed as a 1-way process. Questions are asked of participants and their answers extracted for aggregation and analysis. We show in this work that the process can be reimagined as a bidirectional engagement. Using the IFA, it was possible to both collect data and provide feedback to participants about the growth and development of their child, resulting in improved CHV-facilitated dialog during home visits.

## Appendix

Multimedia Appendix 1 App Ages and Stages Questions and Feedback.

Multimedia Appendix 2 Key indicator tool completed by community-health volunteers in the Information for Action app.

## Abbreviations

ASQ-3: Ages and Stages Questionnaire, Third Edition
CBO: community-based organization
CHEW: community health extension worker
CHV: community health worker
DHMT: District Health Management Team
HSRC: Health Sciences Research Council
ICT: information and communication technology
IFA: Information for Action
LMIC: low- and middle-income country
WHO: World Health Organization
