# Supplementary material for: App-Supported Promotion of Child Growth and Development by Community Health Workers in Kenya: Feasibility and Acceptability Study
Source: JMIR Mhealth Uhealth. 2017 Dec 5;5(12):e182. doi: 10.2196/mhealth.6911 (PMC5736876; doi:10.2196/mhealth.6911)
Supplement: Multimedia Appendix 1 [file mhealth_v5i12e182_app1.pdf]

Multimedia Appendix 1. App ASQ Questions and Feedback

The items closest to the child's age (in months) was automatically selected by the app. The following coding was used by the app to decide on the most appropriate next item. 0=Not Yet; 1=Sometimes; 2=Yes. If Communication 1 scored 2 move to next domain. If scored 1, ask item 2. If item two is asked or item 1 is scored 0 move to feedback 1. This algorithm was repeated for all remaining domains.

AGE (MONTHS)	COMMUNICATION 1	COMMUNICATION 2	GROSS MOTOR 1	GROSS MOTOR 2	FEEDBACK 1	FEEDBACK 2
2	Does your child smile when you talk to him/her?	After you've been out of sight, does your baby smile or get excited when he/she sees you?	When your baby is on his/her back, does she kick her legs?	While your baby is on his/her back, does he/she wave his/her arms, wiggle and squirm?	PLAY. Provide ways for your child to see, hear, feel, move freely, and touch you. Slowly move colourful things for your child to see and reach for. Sample toys: shaker rattle, big ring on a string. Be sure to 1) Give your child affection and show your love 2) Be aware of your child's interests and respond to them 3) Praise your child for trying to learn new skills.	COMMUNICATE. Smile and laugh with your child. Talk to your child. Get a conversation going by copying your child's sounds or gestures. Be sure to 1) Give your child affection and show your love 2) Be aware of your child's interests and respond to them 3) Praise your child for trying to learn new skills.
4	Does your baby make high pitched squeals?	After you've been out of sight, does your baby smile or get excited when he/she sees you?	When you hold your baby in a sitting position, does he/she hold his/her neck steady?	When your baby is on his/her tummy, does she hold her head up, looking around (maybe resting on his/her arms)?	PLAY. Provide ways for your child to see, hear, feel, move freely, and touch you. Slowly move colourful things for your child to see and reach for. Sample toys: shaker rattle, big ring on a string. Be sure to 1) Give your child affection and show your love 2) Be aware of your child's interests and respond to them 3) Praise your child for trying to learn new skills.	COMMUNICATE. Smile and laugh with your child. Talk to your child. Get a conversation going by copying your child's sounds or gestures. Be sure to 1) Give your child affection and show your love 2) Be aware of your child's interests and respond to them 3) Praise your child for trying to learn new skills.
6	If you call your baby when you are out of sight, does he/she look in the direction of your voice?	Does your baby make sounds like "da", "ga", "ka" and "ba"?	if you hold both his/her hands, just to balance your baby, does he/she support his/her own weight while standing?	Does your baby roll from his/her back to his/her tummy, getting both arms out from under him/her?	PLAY. Give your child clean, safe household things to handle, bang, and drop. Sample toys: containers with lids, metal pot and spoon. Be sure to 1) Give your child affection and show your love 2) Be aware of your child's interests and respond to them 3) Praise your child for trying to learn new skills	COMMUNICATE. Respond to your child's sounds and interests. Call the child's name, and see your child respond. Be sure to 1) Give your child affection and show your love 2) Be aware of your child's interests and respond to them 3) Praise your child for trying to learn new skills.
