# Supplementary material for: App-Supported Promotion of Child Growth and Development by Community Health Workers in Kenya: Feasibility and Acceptability Study
Source: JMIR Mhealth Uhealth. 2017 Dec 5;5(12):e182. doi: 10.2196/mhealth.6911 (PMC5736876; doi:10.2196/mhealth.6911)
Supplement: Multimedia Appendix 2 [file mhealth_v5i12e182_app2.pdf]

Multimedia Appendix 2. Key indicator tool completed by CHVs in the IFA app

	**Question**	**Response**	**Key message**
1.	Do you know your child's weight?	Yes	Very good! Weighing your child's regularly is important to know how your child is growing. The nurse at the health facility tells you how you can ensure your child continues to grow well. Please continue going to a health facility to weigh your child.
		No	Weighing your child's regularly is important to know how your child is growing. Please go to a health facility to weigh your child. The nurse at the health facility will also tell you how you can ensure your child continues to grow well.
2.	Can I see the child's immunization card? Is your child up to date with all its immunizations?	Yes	Very good—congratulations! However, you still need to go to the health facility to see the nurse/doctor to make sure your child is growing well. Please go there as soon as possible.
		No	Your child needs to get all the required immunizations. This will protect her/him from diseases, as well as protect her/his friends. Please go to the health facility to see the nurse/doctor as soon as possible. Continue seeing the nurse/doctor even after you get all immunizations to ensure your child is growing well.
4.	Have women and children in the house slept under an insecticide-treated net (ITN) in the last 24 hours?	Yes	Very good. It is important for children to sleep under a net, as they can get malaria if they do not sleep under a net. Malaria is one of the biggest killers of children in our communities. Pregnant women who get malaria are at higher risk of dying in pregnancy. Malaria in pregnancy can also affect the health of the unborn child.
		No	It is important for children to sleep under a net, as they can get malaria if they do not sleep under a net. Malaria is one of the biggest killers of children in our communities. Pregnant women who get malaria are at higher risk of dying in pregnancy. Malaria in pregnancy can also affect the health of the unborn child. If you need to buy a net, please go to the health facility to ask where you can go so.
5.	Does everyone in the household wash their hands with soap every	Yes	Very good. Washing your hands with soap is important, as it kills germs that cause diseases. You should wash your hands with soap when you

	Question	Response	Key message
	time they come into the house from outside and after they use the toilet?		return to the house from outside and after you use the toilet. You should also wash your hands before handling a baby and after you have cleaned a baby. You need to wash your hands before you touch food or drinking water.
		No	Washing your hands with soap is important, as it kills germs that cause diseases. You should wash your hands with soap after you return to the house from outside and after you use the toilet. You should also wash your hands before handling a baby and after you have cleaned a baby. You need to wash your hands before you touch food or drinking water.
6.	In the last 24 hours, did the women and children aged between 6 months and 5 years in the household eat foods from the 4 food groups: (1) starches like ugali, chapattis, bread, rice, potatoes, sweet potatoes; (2) vegetables or fruits; (3) proteins like meat, fish, chicken, omena, milk, eggs, beans, green grams; (4) sugar, oil, or fats like Blue Band?	Yes	Very good. You are eating a balanced diet that contains foods from the 4 food groups. Starches give you energy, vegetables and fruits protect you against diseases, proteins help build your body, and fats and sugar are concentrated sources of energy. However, do not eat too much fats and sugar. Also, while starches are important, you need to add vegetables, fruits, and foods rich in proteins to your diet. If you find it hard to obtain these foods, ask your CHW how you can start a kitchen garden and keep poultry and livestock to provide you with vegetables, fruits, and foods rich in protein. If you are HIV negative, you should breastfeed your child until your child is 2 years old, while providing a balanced diet of solid food. If you are HIV positive, you should breastfeed your child until your child is 1 year old, while providing a balanced diet of solid food. For the first 6 months after birth, a child should be given only breast milk.
		No	You are not eating a balanced diet that contains foods from the 4 food groups. It is important to eat a balanced diet. Starches give you energy, vegetables and fruits protect you against diseases, proteins help build your body, and fats and sugar are concentrated sources of energy. However, do not eat too much fats and sugar. Also, while starches are important, you need to add vegetables, fruits, and foods rich in proteins to your diet. If you find it hard to obtain these foods, ask your CHW how you can start a kitchen garden and keep poultry and livestock to provide you with vegetables, fruits, and foods rich in protein. If you

	Question	Response	Key message
			are HIV negative, you should breastfeed your child until your child is 2 years old, while providing a balanced diet of solid food. If you are HIV positive, you should breastfeed your child until your child is 1 year old, while providing a balanced diet of solid food. For the first 6 months after birth, a child should receive be given only breast milk.
7.	When a woman in the household was last pregnant, did she go to the clinic for antenatal care and did she give birth in a health facility?	Yes	Very good. It is important for a pregnant woman to go to the clinic at least 4 times during her pregnancy. The pregnant woman should go to the clinic as soon she misses her period without waiting for her pregnancy to show. At the clinic, the nurse or doctor can make sure that both the woman and her unborn baby are healthy. At the clinic, the nurse or doctor can also prescribe medicines or treatment if the woman and her unborn baby are not healthy. It is also important to give birth at a health facility, so that both the woman and her child can receive proper care. Women and their families should save money for traveling to birth in a health facility.
		No	It is important for a pregnant woman to go to the clinic at least 4 times during her pregnancy. The pregnant woman should go to the clinic as soon she misses her period without waiting for her pregnancy to show. At the clinic, the nurse or doctor can make sure that both the woman and her unborn baby are healthy. At the clinic, the nurse or doctor can also prescribe medicines or treatment if the woman and her unborn baby are not healthy. It is also important to give birth at a health facility, so that both the woman and her child can receive proper care. Women and their families should save money for traveling to birth in a health facility.
8.	Do you hit or yell at your child when you think she/he has done something wrong?	Yes	Hitting or yelling at your child can create problems with your child’s development. Consider positive parenting practices such as warning your child to not engage in bad behaviors, not allowing her/him to do something unless she/he listens to you, or having both yourself and your child take a few minutes to cool down before talking.
		No	It is good that you do not hit or yell at your child. Hitting or yelling at your child can create problems with your child’s development. Continue positive parenting practices such as warning your child to

	Question	Response	Key message
			not engage in bad behaviors, not allowing her/him to do something unless she/he listens to you, or having both yourself and your child take a few minutes to cool down before talking.
9.	Does your child behave, act, or look different from other children of her/his age?	Yes	Please go to the health facility and have the nurse/doctor take a look your child. Please do not hide your children if you feel they are different from other children.
		No	It is very good that you feel your child is behaving, acting, and looking like other children of her/his age. However, you should take your child to the health facility regularly—even when she/he is not sick—to ensure your child is growing properly.
